# Convergent Hybrid Ablation Versus Catheter Ablation in Patients With Persistent Atrial Fibrillation and Heart Failure: HALT AF Study Protocol and Rationale for a Multicenter Randomized Controlled Trial

**DOI:** 10.1161/JAHA.125.047774

**Published:** 2026-05-29

**Authors:** Omar Ahmed, Elijah R. Behr, Sarah White, Aziz Momin, Riyaz A. Kaba

**Affiliations:** ^1^ Cardiovascular and Genomics Research Institute,School of Health and Medical Sciences City St George’s, University of London Cranmer Terrace London SW17 0RE UK; ^2^ St George's University Hospitals NHS Foundation Trust Blackshaw Road London UK; ^3^ Ashford and St Peter's Hospital Foundation Trust Guildford Road Surrey UK; ^4^ Department of Biological Sciences, Royal Holloway University of London London UK

**Keywords:** catheter ablation, convergent hybrid ablation, heart failure, hybrid ablation, left atrial appendage exclusion, persistent atrial fibrillation, randomized controlled trial, Atrial Fibrillation, Heart Failure, Quality and Outcomes, Arrhythmias, Electrophysiology

## Abstract

**Background:**

The coexistence of persistent atrial fibrillation (AF) and heart failure with reduced left ventricular ejection fraction is associated with greater morbidity and death than either condition alone. Catheter ablation improves outcomes in selected patients, yet results in persistent AF remain unsatisfactory. Convergent hybrid ablation may offer more durable rhythm control by integrating epicardial and endocardial techniques to achieve homogeneous, transmural pulmonary vein and posterior wall isolation. However, randomized data comparing ablation strategies in patients with reduced left ventricular ejection fraction are lacking, leaving a critical evidence gap.

**Methods:**

HALT AF (Hybrid Ablation for Atrial Fibrillation With Heart Failure) is an investigator‐initiated, multicenter, prospective, open‐label, randomized controlled trial with blinded outcome assessment. A total of 120 patients with nonparoxysmal AF, left ventricular ejection fraction <50%, and at least moderate left atrial dilatation undergoing first‐time ablation will be randomized 1:1 to hybrid ablation or catheter ablation. All patients will receive preablation guideline‐directed optimization and undergo strategically equivalent ablation (pulmonary vein isolation plus posterior wall isolation). The primary efficacy end point is freedom from recurrent arrhythmia >30 seconds at 12 months and off class I/III antiarrhythmic drugs, excluding a 3‐month blanking period. The primary safety end point is major adverse cardiovascular events within 30 days. Secondary end points assess cardiac remodeling, biomarkers, symptoms, and quality of life.

**Conclusions:**

HALT AF will be the first randomized trial to compare convergent hybrid ablation and catheter ablation in patients with persistent AF and reduced left ventricular function. By enrolling only patients with reduced left ventricular ejection fraction and dilated atria and by equating lesion sets, HALT AF is designed to provide critical evidence to inform rhythm‐control strategies and guideline recommendations in this high‐risk, underrepresented population.

**REGISTRATION:**

URL: https://www.clinicaltrials.gov; Unique identifier: NCT05411614.

**Nonstandard Abbreviations and Acronyms**
AAD: antiarrhythmic drug
CA: catheter ablation
CABANA: Catheter Ablation Versus Antiarrhythmic Drug Therapy for Atrial Fibrillation
CAPLA: Catheter Ablation for Persistent Atrial Fibrillation
CASTLE‐AF: Catheter Ablation Versus Standard Conventional Treatment in Patients With Left Ventricular Dysfunction and Atrial Fibrillation
CEASE‐AF: Combined Endoscopic Epicardial and Percutaneous Endocardial Ablation Versus Repeated Catheter Ablation in Persistent and Longstanding Persistent Atrial Fibrillation
CONVERGE: Convergence of Epicardial and Endocardial Ablation for the Treatment of Symptomatic Persistent Atrial Fibrillation
EAM: electroanatomic mapping
HA: hybrid ablation
HALT AF: Hybrid Ablation for Atrial Fibrillation With Heart Failure
HARTCAP‐AF: Hybrid Versus Catheter Ablation in Persistent Atrial Fibrillation
ISRCTN: International Standard Randomized Controlled Trial Number
LAA: left atrial appendage
MACE: major adverse cardiac event
PFA: pulsed‐field ablation
PVI: pulmonary vein isolation
PWI: posterior wall isolation
RFA: radiofrequency ablation
T_0_
: harmonized time point

Ablation of atrial fibrillation (AF) in patients with impaired left ventricular ejection fraction (LVEF) carries a class I guideline recommendation for restoring sinus rhythm, particularly where the arrhythmia is a driver of left ventricular (LV) dysfunction.^1^, ^2^ In contrast with patients with preserved LVEF, AF ablation in such patients can reduce heart failure (HF) hospitalizations and prolong survival,^3^ even when HF is advanced.^4^ In such cases, AF ablation is uniquely positioned as a disease‐modifying therapy with the potential to alter the clinical trajectory of both conditions. However, when AF is persistent, outcomes from catheter ablation (CA), which can prove successful in 80% of patients with paroxysmal AF, are unsatisfactory, with only 35% to 50% of patients with nonparoxysmal AF maintaining sinus rhythm after a single procedure.^5^, ^6^ Multiple procedures and adjunctive antiarrhythmic drugs (AADs) may be required, and the clinical trajectory of patients with persistent AF and HF is often one of refractory symptoms, repeated hospitalizations, and steady decline with escalating burden to patients, carers, and health care services.^7^

Mechanistic insights suggest that as AF becomes persistent, the arrhythmogenic substrate extends beyond the pulmonary veins (PVs).^8^ Experimental and human studies demonstrate that increasing AF duration is associated with progressive left atrial (LA) structural remodeling, including interstitial fibrosis, myocyte hypertrophy, and chamber dilatation,^9^, ^10^, ^11^, ^12^ alongside electrophysiological changes such as conduction heterogeneity, shortening of the action potential and effective refractory period, and alterations in ion channel expression and distribution.^11^, ^13^, ^14^ High‐density endocardial and epicardial mapping studies in persistent AF have further identified extra‐PV focal sources, rotational activity, and regions of endocardial–epicardial electrical dissociation, particularly within the posterior LA, suggesting that arrhythmia perpetuation may be sustained by a 3‐dimensional substrate rather than surface triggers alone.^12^, ^15^, ^16^, ^17^

Against this evolving understanding of atrial substrate remodeling, pivotal randomized trials have evaluated a wide variety of pulmonary vein isolation (PVI) plus endocardial‐only anatomic and electrogram‐guided ablation strategies.^18^, ^19^, ^20^, ^21^, ^22^, ^23^, ^24^ However, the most consistent finding has been the absence of incremental benefit beyond PVI alone. In contrast, strategies that incorporate epicardial substrate modification, either alone or as part of a hybrid ablation approach, have shown promise. Thoracoscopic ablation,^24^ thoracoscopic hybrid ablation,^25^, ^26^, ^27^ vein of Marshall ablation,^28^, ^29^ and convergent hybrid ablation (HA)^30^ have all demonstrated superiority over CA in the rhythm control of patients in persistent AF. In recognition, guidelines have elevated thoracoscopic and hybrid (including convergent) ablation strategies from class IIb to class IIa recommendations.^1^ However, despite the clear advantages of early and durable restoration of atrioventricular synchrony,^31^ patients with both persistent AF and reduced LVEF are underrepresented in trials assessing more advanced AF ablation approaches. No randomized trial has specifically evaluated posterior–pericardioscopic convergent HA in patients with reduced LVEF. In CONVERGE (Convergence of Epicardial and Endocardial Ablation for the Treatment of Symptomatic Persistent AF), patients with LVEF <40% were excluded, and the mean baseline LVEF in the hybrid arm was 55.3±7.8%, indicating predominantly preserved systolic function.^30^ Similarly, CEASE‐AF (Combined Endoscopic Epicardial and Percutaneous Endocardial Ablation Versus Repeated Catheter Ablation in Persistent and Longstanding Persistent Atrial Fibrillation), which randomized patients to thoracoscopic HA, enrolled patients with largely preserved LVEF (mean 58.1±8.8%) and excluded those with LVEF <30%.^26^ Similar trends are observed in catheter‐based ablation trials. In CABANA (Catheter Ablation Versus Antiarrhythmic Drug Therapy for Atrial Fibrillation), only 15.2% of participants had HF listed descriptively as a comorbidity in their medical history, and among those with available echocardiographic data, <10% had LVEF <40%.^32^ In CAPLA (Catheter Ablation for Persistent Atrial Fibrillation), patients with LVEF <50% constituted 29% of the cohort, with a median LVEF of 56% (interquartile range, 46%–60%).^22^ Even in CASTLE‐AF (Catheter Ablation Versus Standard Conventional Treatment in Patients With Left Ventricular Dysfunction and Atrial Fibrillation), which enrolled only patients with reduced LVEF,^3^ 30% of patients randomized to CA had paroxysmal rather than persistent AF.

Collectively, the data highlight the underrepresentation of patients with both persistent AF and impaired LV systolic function. Such patients remain disproportionately affected by the lack of a defined ablation strategy, and there remains a critical, unmet need to treat them more effectively.^1^ HALT AF (Hybrid Ablation for Atrial Fibrillation With Heart Failure) is the first randomized trial designed to address this gap, aiming to assess the superiority of convergent HA over CA in patients with persistent AF and LVEF <50%.

As a strategy, the goal of convergent HA is to effectively and categorically isolate the PVs and posterior wall (PW). The technique consists of 2 stages: (1) minimally invasive posterior–pericardioscopic surgical epicardial ablation (targeting the PW and accessible PVs), followed by (2) catheter percutaneous endocardial ablation (ablating areas inaccessible epicardially due to pericardial reflections). The procedures may be performed concurrently (epicardial ablation followed by endocardial ablation during a single admission) or during 2 separate admissions (usually a few months apart), depending on program structure, patient factors, and logistical considerations.^33^ To date, 1 randomized controlled trial has assessed the impact of the strategy in patients with persistent AF. The landmark CONVERGE trial, published in 2020, demonstrated improved 12‐month arrhythmia‐free survival with convergent HA compared with CA in patients with persistent and longstanding persistent AF.^30^ However, ablation strategies were not equivalent (PVI plus posterior wall isolation [PWI] versus PVI alone), and randomization used a 2:1 HA:CA design. These factors limit direct extrapolation to patients with reduced LVEF and underscore the need for a strategy‐level comparison using equivalent electrophysiological procedural end points in an HF population. Only 1 retrospective study has reported outcomes of convergent HA in patients with reduced LVEF and suggested a potential benefit; however, the findings are limited by a small sample size (43 patients) and an observational, nonrandomized study design.^34^

To determine whether patients with an unmet, clinically critical need for more effective AF ablation may benefit from convergent HA, we designed the HALT AF study. HALT AF will test the hypothesis that convergent HA improves clinical outcomes in patients with persistent AF and reduced LVEF undergoing first‐time AF ablation, compared with CA, and will provide valuable randomized evidence for its use in these patients. By comparing strategically equivalent lesion sets (PVI and PWI) and incorporating contemporary epicardial and endocardial ablation techniques with blinded outcome assessment, it aims to provide robust, clinically relevant results with high validity. Furthermore, it will provide pivotal data on the safety and efficacy of HA and CA in patients with reduced LVEF, adding valuable guideline‐informing evidence in the management of this high‐risk population.

## Methods

### Ethical Approval and Informed Consent

The study is being conducted in accordance with the principles outlined in the Declaration of Helsinki, and formal ethical approval has been obtained from the UK National Health Service Health Research Authority (Reference 21/AW/0082). This trial was registered at ClinicalTrials.gov (NCT05411614) in June 2022, before the first enrollment, and in the ISRCTN (International Standard Randomized Controlled Trial Number) registry (ISRCTN31213485). All participants provided written informed consent.

### Transparency and Openness Statement

In accordance with the American Heart Association Journals' Transparency and Openness Promotion Guidelines, the data and analytic methods supporting this study will be made available from the corresponding author upon reasonable request, and materials used to conduct the research are described in sufficient detail to allow replication.

### Study Design

**Figure 1:**
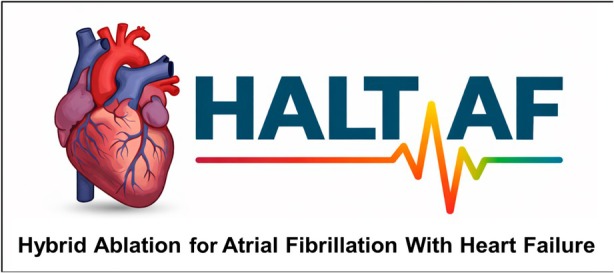
Trial logo for HALT AF. HALT AF indicates Hybrid Ablation for Atrial Fibrillation With Heart Failure.

**Figure 2:**
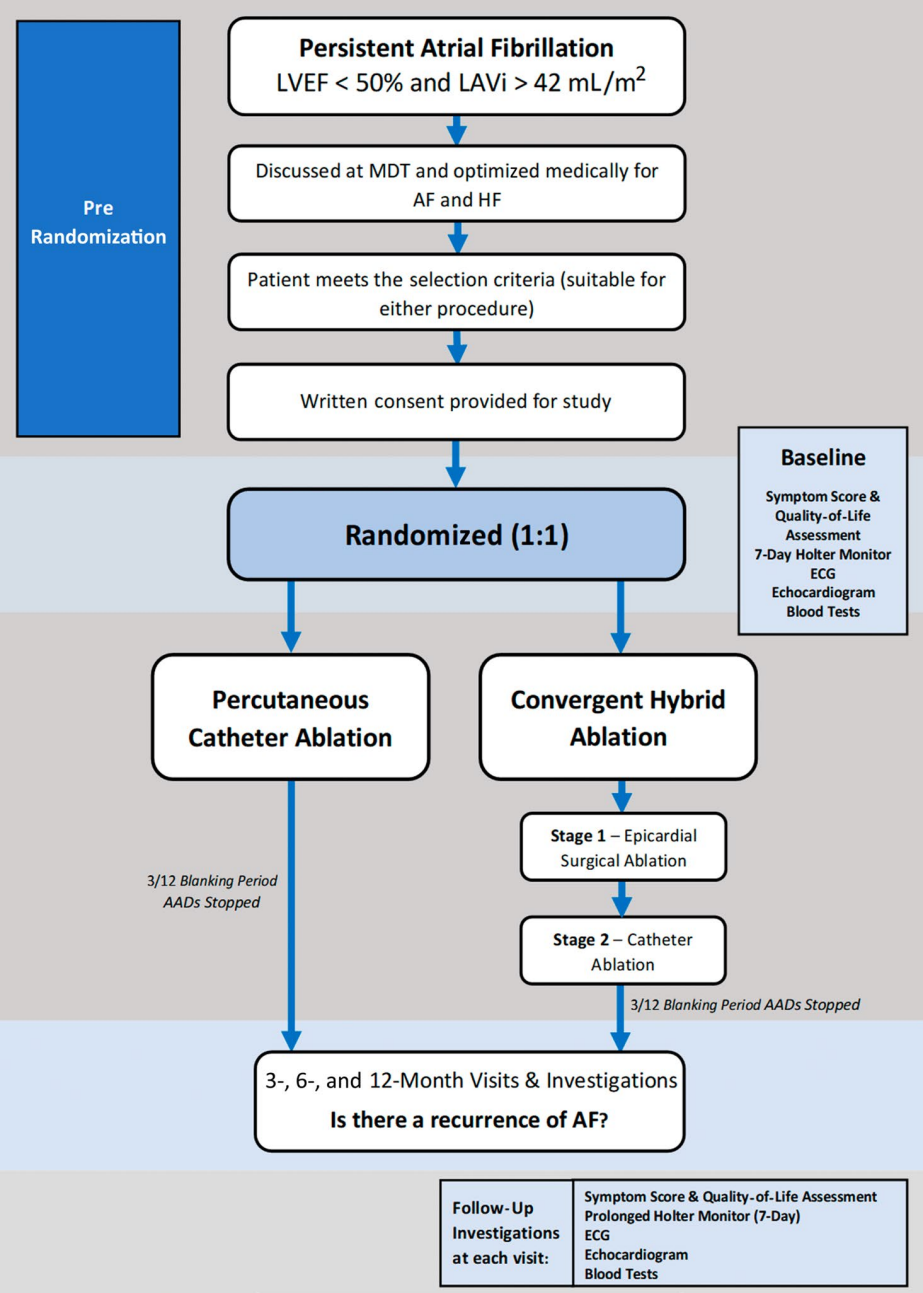
Trial design and flow diagram for HALT AF. AAD indicates antiarrhythmic drug; AF, atrial fibrillation; HF, heart failure; LAVi, left atrial volume index; LVEF, left ventricular ejection fraction; and MDT, multidisciplinary team.

HALT AF is an investigator‐initiated, multicenter, prospective, randomized controlled trial with an open‐label design and blinded evaluation of end point outcomes. Approximately 120 participants from 5 UK centers within the National Health Service will be randomized in a 1:1 ratio to convergent HA (with or without left atrial appendage [LAA] exclusion) or endocardial CA alone. Figure 1 illustrates the trial logo, and Figure 2 reports the patient pathway through the trial.

Before inclusion, potentially eligible participants will be reviewed at a dedicated multidisciplinary team meeting comprising cardiac electrophysiologists, cardiac surgeons, HF experts, imaging consultants, and nurse specialists. All patients will receive optimization of medical and lifestyle factors in accordance with contemporary AF and HF management guidelines.^1^, ^35^, ^36^, ^37^, ^38^ In accordance with best clinical practice, only patients in whom rhythm control remains clinically indicated after optimization will be considered for ablation. Patients will be considered eligible for the study if there is clinical equipoise on the ablation strategy (ie, the patient is suitable for either HA or CA, with neither therapy considered more beneficial). Following confirmation of eligibility and provision of written informed consent, participants will be enrolled.

### Patient Population

#### Inclusion Criteria

1. Age ≥18 years
2. Persistent or long‐standing persistent AF (as defined by current guidelines)^1^
3. LVEF <50%*
4. At least moderate LA dilatation (LA volume index ≥42 mL/m^2^)*
5. Suitable for either procedure (clinical equipoise)

*As measured on transthoracic echocardiography in line with current guidelines.^35^ The LVEF threshold (<50%) is intended to capture both HF with mildly reduced ejection fraction (LVEF 40–49%) and HF with reduced ejection fraction (LVEF <40%). Baseline LVEF will be summarized both as a continuous variable and in prespecified clinically relevant categories (eg, <40% and 40%–49%).

#### Exclusion Criteria

1. Unable to provide written consent
2. Not yet optimized medically for HF or AF
3. Previous open‐heart surgery
4. Active infection; esophageal ulcer, stricture, or varices
5. Prior catheter ablation of AF (prior ablation for right atrial flutter, supraventricular tachycardia, or ventricular tachycardia acceptable)
6. Contraindication to anticoagulation or thrombus in the LA despite anticoagulation
7. Severe valvular heart disease (defined as severity greater than mild)
8. Unstable coronary artery disease
9. Uncontrolled ventricular arrhythmia
10. Myocardial infarction or stroke within 90 days
11. Pregnant, breastfeeding, or women of childbearing age who plan to get pregnant within 6 months
12. Severe concomitant condition or the presence of an implanted device that would preclude the safe performance of trial procedures or protocol‐mandated imaging (eg, a recently implanted atrial septal defect closure device). The presence of a permanent pacemaker, implantable cardioverter‐defibrillator or cardiac resynchronisation therapy device does not constitute an exclusion criterion.

### Randomization and Blinding

**Figure 3:**
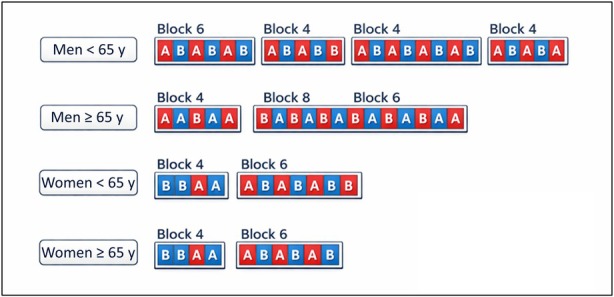
Visual representation of stratified permuted block randomization used in HALT AF. Example block sizes shown are illustrative only. A = hybrid ablation; B = catheter ablation. Block randomization used variable block sizes of 4, 6, and 8 to reduce predictability. HALT AF indicates Hybrid Ablation for Atrial Fibrillation With Heart Failure.

Randomization and data collection will use Research Electronic Data Capture.^39^ Research Electronic Data Capture is a secure, web‐based application that allows for longitudinal data collection with audit trails to ensure data integrity. It will be accessed via a secure server stored within St George's University, London, UK. Once enrolled, a patient identification number will be generated by registering the patient for an electronic case report form. Authorized staff will be allocated a user name and password for this purpose. Once informed consent and eligibility are confirmed, the staff member can enter the patient's details, and the software will randomly assign the subject to a trial arm. Randomization uses stratified permuted block randomization with concealed block sizes to ensure parity and comparability between arms with respect to key baseline characteristics known to influence AF incidence, prevalence, and progression.^40^, ^41^ An independent statistician generated the allocation sequence. Participants will be stratified by sex and age (<65 versus ≥65 years), creating 4 strata (men <65, men ≥65, women <65, and women ≥65 years). Randomization is to be conducted in a 1:1 ratio within each stratum using permuted blocks (Figure 3) of varying sizes (4, 6, and 8). Blinding to treatment is not feasible given the differences between the arms (single‐stage versus 2‐stage). However, follow‐up assessments (ECG interpretation, rhythm monitoring, echocardiography, and blood tests) and end point validation will be performed by independent clinicians or technicians blinded to the treatment allocation. Participants are fully informed of their allocated treatment and receive standard clinical care. To preserve blinding of outcome assessors, participants are asked not to disclose treatment allocation during research assessments only and are explicitly advised that allocation may be disclosed at any time when clinically necessary.

### Treatments

All study interventions will be performed under general anesthesia. In both treatment arms, the objective is to achieve PVI and PWI. In CA, this is conventionally achieved as a single‐stage procedure requiring a single admission. In contrast, HA requires both epicardial (stage 1) and endocardial (stage 2) components to achieve PVI and PWI, as pericardial reflection limits the extent of ablation that may be performed during stage 1. Stage 2 is therefore integral to lesion‐set completion, and neither PVI nor PWI may be completely achieved without it. The stages of HA may be performed during a single admission or across 2 separate admissions, with the latter approach more commonly adopted in contemporary practice.^42^, ^43^ HALT AF adopts a 2‐admission staged approach to enhance procedural feasibility and safety in a high‐risk HF population. This design aligns with current HA patient pathways within the UK and European health care services, and increasingly among centers in the United States, and facilitates coordination among cardiac surgical, electrophysiology, and anesthesia teams, as well as with existing procedural remuneration frameworks. A staged approach limits prolonged single‐anesthetic exposure and allows interval resolution of acute tissue edema before definitive endocardial mapping and completion of isolation. Consequently, the hybrid strategy is associated with a greater up‐front procedural burden than single‐stage CA. This difference in exposure is intrinsic to the strategies being compared and will be measured and reported explicitly. Formal consent for each procedure will be obtained in accordance with standard clinical practice. Each treatment arm is described in detail below.

#### Percutaneous Endocardial‐Only Catheter CA: Comparator Group

CA will be performed under general anesthesia on uninterrupted anticoagulation. Transesophageal echocardiography will be used to exclude LA thrombi. Femoral access will be established using ultrasound guidance. LA access will be via transseptal puncture under fluoroscopic, hemodynamic, and transesophageal echocardiography guidance, with intravenous heparin administered and maintained throughout to achieve an activated clotting time >350 seconds. All CA cases will use electroanatomic mapping (EAM). At the onset of the trial, radiofrequency ablation (RFA) was the key percutaneous AF ablation energy available. To reflect evolving contemporary clinical practice while preserving the strategy‐level comparison, pulsed‐field ablation (PFA) under EAM guidance has been incorporated into the protocol and is permitted for endocardial ablation in both treatment arms, using a pentaspline ablation catheter (Farapulse, Boston Scientific, Marlborough, MA). The introduction of PFA did not alter the mandated procedural end points or lesion‐set objectives. Further details are provided in the Ablation Strategy and Energy Source section. The standard approach to PVI and PWI with PFA has been well described in the literature and prior trials.^44^, ^45^ The prespecified RFA and PFA lesion sets for the CA arm are defined below:

RFA (point‐by‐point RFA guided by ablation‐index or high‐power short‐duration protocols under EAM)

- Antral PVI (wide area circumferential catheter ablation)
- Linear ablation between the contralateral superior veins (“roof line”)
- Linear ablation between the contralateral inferior PVs (to complete a so‐called “box lesion”)
- Mapping and ablation of any atrial tachycardia that occurs during the procedure

PFA (pentaspline PFA catheter with EAM)

- Antral PVI: individual isolation of each PV with a combination of “basket” and “flower” lesions
- At least 2 rows of overlapping lesions across the PW for PWI
- Mapping and ablation of any atrial tachycardia that occurs during the procedure

Isolation will be validated in all cases by assessing local electrograms and performing pacing maneuvers to detect electrical block. For PFA procedures, a postablation EAM will also be created to evaluate for residual electrical activity. Areas of reconnection or gaps will be targeted with supplementary ablation until isolation is complete or deemed impossible to achieve. Procedural details, including energy source, mapping system, lesion sets, ablation duration, confirmation of isolation, and acute procedural outcomes, will be documented prospectively. Acute procedural success will be defined as the achievement of PVI and PWI at the end of the procedure.

#### Convergent HA: Intervention Arm

The approach to convergent HA has been described previously.^46^ HA comprises 2 distinct procedural stages, performed under general anesthesia, scheduled a few months apart. While epicardial ablation at stage 1 contributes to PW and PV substrate modification, complete PVI and PWI can only be confirmed following stage 2, owing to the anatomic limitations imposed by the pericardial reflections. In addition to epicardial ablation at stage 1, video‐assisted thoracoscopic surgical LAA exclusion may be offered as a supplementary nonablation procedure for thromboembolic risk management. LAA exclusion is not protocol mandated and does not form part of the ablation lesion set. It will be performed only when clinically indicated for thromboembolic risk management and is not intended to influence rhythm outcomes. Where performed, its use will be prospectively recorded and reported descriptively. For completeness, both components are reported below.

##### Stage 1a: Epicardial Ablation of the PW and PVs

Stage 1 HA is performed under general anesthesia, with preprocedure transesophageal echocardiography performed to exclude LA thrombus. Epicardial ablation is performed via a subxiphoid incision using a dedicated linear vacuum‐assisted irrigated unipolar RFA catheter (Epi‐Sense System; AtriCure, Mason, OH). The ablation catheter is inserted into the posterior pericardial space via a cannula. The focus of epicardial ablation is the PW. At least 2 rows of sequential and overlapping linear lesions will be delivered across the PW, with visual inspection to ensure homogeneous coverage of all accessible epicardial PW regions. Ablation will be performed under continuous esophageal temperature monitoring to ensure safety. Following PW ablation, the caudal aspects of both PVs will be ablated up to the limits of the pericardial reflections. The pericardial reflections will not be dissected. Once ablation is completed, the incision will be closed. No pleural drains are required to be left in situ following stage 1a.

##### Stage 1b (Not Mandated): Thoracoscopic LAA Exclusion

Once epicardial ablation is completed, the subject may proceed to LAA exclusion. LAA exclusion will be performed via a video‐assisted thoracoscopic surgical approach using a 3‐port thoracoscopy (2‐ to 3‐mm port in the second intercostal space in the midclavicular line, a 5‐mm port in the fourth intercostal space along the midaxillary line, and a 12‐mm port in the sixth intercostal space in the posterior axillary line). The left lung will be deflated to visualize the pericardial sac, and the pericardium will be opened using a LigaSure device (Medtronic, Minneapolis, MN). The LAA will be identified and measured using a dedicated AtriCure device before deployment of the AtriClip Pro device. Occlusion at the base of the LAA will be confirmed by transesophageal echocardiography before final device deployment. If LAA exclusion is performed, a drain will be placed in the pleural cavity via the lowest port. Any HA cases where LAA exclusion is performed will be prospectively recorded and reported descriptively.

##### Stage 2: Percutaneous Endocardial CA to achieve PVI and PWI

The endocardial component of the convergent procedure (stage 2) will be performed during a separate admission ≈8 to 16 weeks after stage 1, where possible. The procedure follows the same transseptal approach as the CA‐only procedure. As per the comparator arm, endocardial ablation may use thermal (RFA) or nonthermal (PFA) energy sources. A baseline EAM will be created to assess the ablation performed at stage 1. Supplementary endocardial ablation will be administered to achieve PVI and PWI. Confirmation of block or isolation after ablation will follow the CA arm protocol (including assessment of local electrograms and pacing maneuvers to demonstrate block and, where applicable, creation of a post‐PFA EAM). Any areas of residual electrical activity or gaps will be targeted with supplementary ablation until isolation is confirmed or deemed not technically achievable. As with the CA arm, procedural details, including energy source, mapping system, lesion sets, procedure duration, confirmation of isolation, and acute procedural outcomes, will be recorded prospectively. Acute procedural success of HA will be defined as confirmation of PVI and PWI following stage 2. The endocardial lesion sets for stage 2 HA are specified below:

RFA (point‐by‐point RFA guided by ablation‐index or high‐power short‐duration protocols under EAM guidance)

- Antral PVI (wide area circumferential catheter ablation lesions as needed to complete PVI)
- Linear ablation comprising a complete or partial roof and floor line (as required, depending on ablation at stage 1) to achieve PWI
- Mapping and ablation of any atrial tachycardia that occurs during the procedure

PFA (pentaspline PFA catheter under EAM guidance)

- Antral PVI: individual isolation of each PV (as needed) with a combination of flower and basket lesions
- Overlapping lesions across the PW (as needed) to achieve PWI
- Mapping and ablation of any atrial tachycardia that occurs during the procedure

#### Ablation Strategy and Energy Source

RFA or PFA may be used for endocardial ablation in either treatment arm. While studies have suggested that PFA may enhance procedural efficacy and patient pathways, randomized comparisons and meta‐analyses have not consistently shown it to be superior to thermal ablation in terms of freedom from recurrent arrhythmia.^47^, ^48^, ^49^ Importantly, energy modalities are not the object of comparison in HALT AF. Rather, the trial is designed to compare ablation strategies, namely, epicardial–endocardial ablation versus endocardial‐only ablation, and to determine whether the addition of an epicardial lesion set confers incremental benefit over endocardial ablation alone, irrespective of endocardial energy modality. Accordingly, the protocol mandates identical electrophysiological procedural end points in both arms, comprising confirmation of PVI and PWI, irrespective of the endocardial energy source, with randomization strictly preserved at the strategy level. Acute procedural success, defined as electrophysiological confirmation of PVI and PWI at the conclusion of the procedure, will be prospectively recorded in all cases to verify end point achievement across arms. Ablation energy use will be reported transparently and evaluated in prespecified exploratory analyses to assess whether strategy‐level treatment effects appear consistent across endocardial energy modalities. These analyses are descriptive and hypothesis generating only, and the study is not powered to compare outcomes between PFA and RFA or to support inference about energy‐source superiority or procedural sequencing.

#### Postprocedure Care and Anticoagulation (Both Arms)

All patients will receive standard postprocedural care in accordance with local institutional protocols. Patients will be advised that they will be admitted and monitored overnight for complications. Anticoagulation will resume on the day of the procedure unless clinically contraindicated. Patients who have had a drain sited following LAA exclusion will have it removed, and all patients will be discharged the following day after a focused echocardiogram and on their usual medications, unless there is an indication for a longer admission. Same‐day discharge will be permitted following adequate monitoring and imaging, in accordance with local best‐practice protocols and patient choice. AADs (class I/III) may be continued or initiated in the early postoperative period. However, all class I or class III AADs will be discontinued by 3 months after ablation in both arms (at the end of the blanking period). Patients will be closely followed up postoperatively with early review under the nurse‐led postablation service. All patients will be provided with the research team's contact details and encouraged to contact them early if they notice any potential complications or have concerns.

#### Follow‐Up Schedule

To ensure methodological equivalence across treatment strategies, efficacy follow‐up is anchored to a harmonized time point (T_0_), defined as the date on which each participant has completed the analogous intended lesion set (PVI plus PWI), which represents completion of the randomized ablation strategy. In the CA arm, T_0_ occurs at the index procedure; in the hybrid arm, T_0_ occurs following completion of stage 2. This strategy‐level framework ensures that rhythm outcomes are assessed only after the equivalent lesion set has been delivered in both arms and avoids misclassification due to incomplete or partial procedures. All clinical events occurring between stage 1 and stage 2 in the hybrid arm will be prospectively captured, adjudicated, and included in secondary and safety analyses. Variation in the timing of stage 2, therefore, affects only the scheduling of post‐T_0_ assessments and does not alter the definition of the primary efficacy analysis. This design constitutes a prespecified postcompletion (T_0_‐anchored) efficacy framework and is not intended to estimate treatment effects from the time of randomization. Participants who do not complete stage 2 will remain included in the primary binary efficacy analysis within the modified intention‐to‐treat population. For these participants, T_0_ will be assigned as the protocol‐scheduled stage 2 time point, defined as 3 months after stage 1. Primary end point status will be classified according to the prespecified end point definitions using available postrandomization data on rhythm and AAD use. Noncompletion of stage 2 will be recorded and incorporated into end point adjudication and sensitivity analyses in accordance with the prespecified analysis plan.

The follow‐up schedule and tests are detailed in Table 1. Follow‐up will consist of 3 scheduled reviews (at 3, 6, and 12 months after T_0_). At each visit, clinical and rhythm status will be assessed, and cardiac function will be evaluated at prespecified time points. Scheduled evaluation will include a 12‐lead ECG, a 7‐day ambulatory ECG monitor (at 6 and 12 months), echocardiography (at 6 and 12 months), blood tests, clinical assessment, and quality‐of‐life assessments. Blood tests at baseline and follow‐up will include NT‐proBNP (N‐terminal pro‐B‐type natriuretic peptide), CRP (C‐reactive protein), lipid profile, renal function, and complete blood count. To ensure consistency across centers, each time point will include an allowable visit window. Out‐of‐window assessments will be documented and may contribute to supportive or sensitivity analyses; however, they will not replace the prespecified time point in the primary analysis. Where they document a qualifying recurrence after blanking, they may contribute to end point adjudication, but they do not substitute for scheduled windowed assessment. Further information on the visit plan and data collection is provided in Table 1. Unscheduled follow‐up and investigations may be initiated at any time if symptoms or clinical concerns arise that suggest a complication or recurrence of arrhythmia. The findings will be recorded and included in end point adjudication, but they will not substitute for scheduled visits unless they fall within the prespecified window. All investigations will be conducted by clinicians and technicians blinded to the patient's assigned arm, with analysis performed separately from the clinical encounter and adjudicated blindly.

**Table 1:** Visit Plan and Data Collection Schedule for HALT AF

	Baseline	3‐mo	6‐mo	12‐mo
Visit window (T_0_+),* d	…	90±14	180±21	365± 30
Eligibility criteria	X			
Informed consent	X			
Randomization	X			
Baseline demographics	X			
Optimization of medical therapy for AF and HF	X	X	X	X
Medical history, including medications, symptom score, and need for any admission or rhythm control	X	X	X	X
Clinical examination	X	X	X	X
12‐lead ECG	X	X	X	X
Blood tests†	X	X	X	X
Echocardiography	X		X	X
EQ‐5D‐5L	X		X	X
MLHFQ	X		X	X
AFEQT	X		X	X
Adverse event review		X	X	X
7‐d Holter monitor			X	X

AF indicates atrial fibrillation; AFEQT, Atrial Fibrillation Effect on Quality‐of‐Life Questionnaire; EQ‐5D‐5L, EuroQol 5‐Dimension 5‐Level questionnaire; HALT AF, Hybrid Ablation for Atrial Fibrillation With Heart Failure; HF, heart failure; MLHFQ, Minnesota Living With Heart Failure Questionnaire; and T_0_, harmonized time point.

* T_0_ = completion of the intended lesion set: pulmonary vein isolation and posterior wall isolation.

^†^ Blood tests include N‐terminal pro‐B‐type natriuretic peptide, C‐reactive protein, renal function, complete blood count, and lipid profile.

#### Blanking Period

The blanking period extends from T_0_ to 90 days after the procedure. Arrhythmias during the blanking period will be recorded but will not count toward failure of the primary end point. Blanking‐period arrhythmias will not reset follow‐up or affect end point timing.

#### Management of Arrhythmia Recurrence

Management of recurrent arrhythmias will be in accordance with standard clinical care. A recurrence of arrhythmia within the 3‐month blanking period will be managed with medical therapy and electrical cardioversion as indicated. Repeat ablation is not clinically indicated nor permitted during the blanking period. Any recurrence during the blanking period will be documented but will not constitute a failure of the primary outcome. Arrhythmia recurrence occurring after the blanking period will be managed by optimizing medications for AF and HF, admission if required, and rhythm control via external cardioversion and repeat or “redo” ablation as indicated. Electrical cardioversion may be undertaken for symptomatic atrial arrhythmia or hemodynamic compromise, and all cardioversions will be recorded with the rhythm documentation supporting the indication.

Percutaneous CA will be the primary repeat procedure for patients in both treatment arms and will be discussed and offered to all patients with recurrence, as clinically indicated. As a general principle, redo ablation should be supported by objective documentation of rhythm and the persistence of arrhythmia despite appropriate medical therapy, unless urgent intervention is clinically required. The decision to proceed with repeat ablation will be documented, including evidence of rhythm, symptom burden, and relevant clinical factors, with all such events recorded and incorporated into end point classification to minimize center‐level bias. Repeat epicardial AF ablation is not indicated for any patient who has already undergone stage 1 HA, given the risks of pericardial adhesions and repeat epicardial access following the index procedure. Epicardial ablation (stage 1 HA) may be offered to patients in the CA arm (crossover) if AF recurrence persists despite a primary redo procedure following a further multidisciplinary team discussion. A repeat ablation of any type will be considered as treatment failure for the 12‐month binary primary rhythm end point. Repeat ablation will not reset the timing of the primary time‐to‐event analysis, which is defined as the time to the first documented recurrence. Rates and timing of cardioversion and repeat ablation will be reported as secondary outcomes.

### Efficacy End Points

#### Primary End Point

The primary efficacy end point is freedom from any atrial arrhythmia (defined as AF, atrial flutter, or atrial tachycardia lasting >30 seconds) off class I or III AADs through 12 months after T_0_ (completion of the intended ablation lesion set). The primary end point is evaluated from the end of a 3‐month blanking period. Recurrence may be detected on any of the following methods during scheduled or unscheduled symptom‐driven encounters and is considered met on first documentation, irrespective of frequency or symptom status.

- 12‐lead ECG
- Rhythm strip
- 7‐day Holter monitor (or any Holter >24 hours if arranged via usual clinical care)
- Device interrogation (where applicable)

For participants who complete the intended ablation lesion set, follow‐up timing is anchored to T_0_. Participants who undergo partial delivery of their allocated ablation strategy but are unable to complete the intended lesion set for any reason (including persistent LA thrombus precluding hybrid stage 2 or procedural complications) will remain included in the primary efficacy analysis within the prespecified modified intention‐to‐treat analysis set and end point definitions. All interstage events and procedural exposures in the hybrid arm will be prospectively captured within safety and secondary analyses. For the 12‐month binary rhythm end point, these participants will be classified on the basis of their observed rhythm status, antiarrhythmic drug use, cardioversion, and repeat ablation status, using the same end point definitions as for participants who reach T_0_. Cardioversion or repeat ablation for arrhythmia recurrence occurring after the blanking period will be considered a treatment failure for the binary rhythm end point.

Patients who demonstrate no recurrence of qualifying arrhythmia and do not require cardioversion or repeat ablation after the blanking period are considered treatment successes with respect to efficacy.

#### Secondary and Exploratory End Points

Secondary and exploratory end points will use data from investigations evaluated over the 12‐month follow‐up and are listed below:

1. Freedom from any atrial arrhythmia lasting >30 seconds after a single completed procedure on class I/III AADs.
2. Freedom from atrial arrhythmias after any redo procedures (on or off class I or III AADs). This end point accounts for the overall success rate when additional ablations are performed.
3. LV structural remodeling, as measured by change in LVEF in response to either procedure (from pre‐ to postablation echocardiography up to 12 months).
4. LA remodeling in response to either technique (as measured from pre‐ to postablation echocardiography up to 12 months).
5. Change in NT‐proBNP and prespecified circulating biomarkers (including CRP, lipid profile, renal function, and complete blood count) from baseline up to 12 months.
6. To evaluate the effects of the interventions on symptoms, as assessed by change in the modified European Heart Rhythm Association score and New York Heart Association functional class from baseline up to 12 months after ablation.
7. To evaluate the effects of the interventions on quality of life, as assessed by change (from baseline) in 3 highly validated quality‐of‐life scores:
  - European Quality 5 Dimensions 5‐Level scale^50^
  - AF Effect on Quality‐of‐Life Questionnaire^51^
  - Minnesota Living With Heart Failure Questionnaire^52^

#### Safety End Points

All complications occurring during the trial period will be recorded and reported. A list of expected complications is reported in Table 2, comprising adverse events and serious adverse events. The primary safety end point is defined as the incidence of major adverse cardiac events (MACEs) within 30 days of any procedure (or part of a procedure). MACEs are a widely reported composite end point in interventional and surgical cardiac trials and have been used to report outcomes in HA.^53^, ^54^ HALT AF will incorporate the 5 key components of MACEs. All procedure‐related complications will be recorded and categorized as early (within 30 days) or late (>30 days). The primary safety analysis focuses on early (30‐day) MACE rates across arms. Secondary safety outcomes are defined as the incidence of any procedural or non–procedure‐related adverse events through 12 months in each study arm.

**Table 2:** Adverse and Serious Adverse Events Expected in HALT AF

Adverse events	Serious adverse events
Bruising, hematoma, vascular injury not requiring intervention	Vascular complications requiring blood transfusion or intervention
Pericardial or pleural effusion (requiring observation only)	Symptomatic pleural or pericardial effusion requiring intervention
Pneumothorax requiring observation	Pneumothorax requiring chest drain
Chest infection or pneumonia	Empyema
Pulmonary edema	Myocardial infarction
Temporary phrenic nerve injury	Permanent phrenic nerve injury
Localized soft‐tissue infection	Stroke or transient ischemic attack
Periprocedural ventricular arrhythmia	Pulmonary vein stenosis (>70% reduction in baseline diameter)
	Procedure‐related requirement for permanent pacemaker insertion
	Cardiac trauma requiring surgical intervention
	Atrio‐esophageal fistulae
	Radiation‐induced skin injury
	Death

HALT AF indicates Hybrid Ablation for Atrial Fibrillation With Heart Failure.

#### Key MACE Composite Components Used in HALT AF

1. Acute myocardial infarction
2. Stroke or transient ischemic attack
3. Cardiovascular death or any procedure‐related death
4. Unstable angina requiring urgent intervention
5. Heart failure (new or worsening)

### Data and Safety Monitoring Board

The trial will be monitored independently, with annual review by an independent board that will retain oversight of the study, with additional ad hoc meetings convened as needed or if emerging safety signals or investigator concerns arise. The board will consist of at least 3 senior experts in trial methodology and trial management who are unaffiliated with the sponsor or the trial. At each review, trial progress, protocol adherence, completeness of follow‐up, and all adverse events will be discussed. In parallel, the sponsor will conduct annual monitoring of regulatory compliance, governance adherence, and completeness of safety reporting.

### End Point Adjudication

Arrhythmia end points will be adjudicated by independent clinical specialists (clinicians and physiologists) who are not involved in the delivery of the index procedure. Rhythm recordings (including ECGs, Holter monitors, and device interrogations where available) will be reviewed retrospectively and independently of the clinical encounter, with reviewers blinded to treatment allocation, procedural strategy, and clinical outcomes. In cases of disagreement or if outcomes remain undefined, consensus or a further independent clinician will be used to establish rhythm end points. Similarly, all echocardiographic analyses are to be performed by experienced readers blinded to the allocated treatment. Image acquisition will follow a standardized protocol with images anonymized at the point of acquisition and assigned coded identifiers to prevent reviewers from inferring group allocation. Measurements will be adjudicated independently by 2 observers, with discrepancies resolved by consensus or, when necessary, by a third senior reviewer. Inter‐ and intraobserver variability are to be assessed periodically in a random sample of studies.

### Statistical Analyses

The primary efficacy outcome will be analyzed using a modified intention‐to‐treat approach, including all randomized patients who undergo any component of their allocated ablation strategy. This approach was prespecified to reflect the pragmatic strategy‐based design of HALT AF, recognizing that the trial evaluates procedural strategy effectiveness and that patients without procedural exposure do not enter the risk set for procedural efficacy. Patients randomized but not exposed to any procedural component will be described separately and excluded from efficacy analyses. Participants who undergo partial delivery of the allocated strategy but are unable to complete the intended lesion set for any reason will remain in the primary efficacy analysis and will be handled in accordance with the prespecified end point rules. As a conservative sensitivity analysis, patients who are randomized but not exposed to any procedural component will be classified as treatment failures for the 12‐month binary end point.

#### Primary Efficacy Analysis

The primary end point is freedom from any documented atrial arrhythmia (AF, atrial flutter, atrial tachycardia) lasting >30 seconds between the end of the 3‐month blanking period and 12 months. The proportions of patients free from recurrent atrial arrhythmia lasting >30 seconds off AADs at 12 months after ablation, within the prespecified assessment window (T_0_+365±30 days), will be compared between treatment groups using Poisson regression with robust variance to estimate adjusted risk ratios and risk differences, with 95% CIs. The primary adjusted model will include the randomization strata (age category and sex). Poisson regression with robust variance was chosen to directly estimate risk ratios for this binary outcome. Unadjusted comparisons will be performed using the χ^2^ or Fisher's exact test, as appropriate. A time‐to‐first arrhythmia recurrence analysis will serve as the key supportive analysis. Time will be measured from T_0_ (completion of the intended lesion set) to the first documented recurrence identified on any scheduled or unscheduled rhythm monitoring after the blanking period. Participants without recurrence will be censored at the date of their most recent rhythm assessment or at the end of the prespecified follow‐up window (T_0_+365±30 days), whichever occurs first. Kaplan–Meier survival curves will be constructed, and treatment differences will be evaluated using a Cox proportional hazards model adjusted for randomization strata (age, sex). A prespecified sensitivity model will additionally adjust for baseline LVEF category (<40% and 40%–49%) and AF duration category. Time‐to‐event analyses will be performed among participants who reach T_0_. Participants who do not reach T_0_ will be summarized descriptively and included in sensitivity analyses where appropriate.

#### Primary Safety Analysis

Statistical analyses of the primary safety outcome will be conducted in the as‐treated population, including participants who have received a randomized procedure in whole or in part. Safety outcomes will be summarized descriptively and compared using χ^2^ or Fisher's exact tests, as appropriate. Interstage events in the hybrid arm will be captured and reported transparently as part of the overall safety assessment.

#### Secondary Analyses

Repeated‐measure continuous outcomes, including LVEF, LA volume, biomarkers, and quality‐of‐life scores, will be analyzed using linear mixed‐effects models with the time‐by‐treatment interaction fixed effect indicating the treatment effect. Binary secondary end points (eg, freedom from arrhythmia on antiarrhythmic medication, binary improvement in New York Heart Association class) will be analyzed using models analogous to those used for the primary analysis. Secondary and subgroup analyses are supportive and exploratory.

#### Missing Data

The primary analysis will use all available rhythm data collected according to the prespecified visit schedule, with the 12‐month assessment defined as T_0_+365±30 days. For the binary primary end point, participants will be classified as having recurrence if atrial arrhythmia is documented at any time after the blanking period and within the follow‐up period. Participants with insufficient rhythm data to determine 12‐month recurrence status will be identified, and the extent of missing primary end point data will be reported by treatment arm. Prespecified sensitivity analyses will assess the robustness of the findings under conservative assumptions, including classifying participants with missing end point status as having experienced a recurrence. These analyses are intended to assess the potential impact of missing data on the primary strategy‐level comparison. The frequency and reasons for nonexposure, withdrawal, and loss to follow‐up will be summarized descriptively by treatment arm to assess potential differential attrition.

#### Exploratory and Subgroup Analyses

Exploratory analyses will assess the consistency of treatment effects across prespecified subgroups, including age, sex, baseline LVEF category (eg, <40% and 40%–49%), AF duration, LA size, body mass index, and HF pathogenesis. Treatment‐by‐subgroup interactions will be explored using regression models and summarized graphically. Endocardial energy modality (RFA or PFA) and LAA exclusion status will be reported by treatment arm, and exploratory analyses will assess whether strategy‐level treatment effects appear consistent across these factors. These analyses are descriptive and hypothesis generating, and no formal inference about the superiority of any energy source is intended.

#### Per‐Protocol Analysis

A per‐protocol analysis will be performed as a secondary sensitivity analysis. The population will include patients who received their allocated ablation strategy and completed the intended lesion set (PVI plus PWI), with no significant protocol deviations. All protocol deviations will be predefined and reported. Per‐protocol analyses will mirror the primary efficacy analyses.

#### Statistical Threshold

All tests will be 2‐sided, with a *P* value <0.05 considered statistically significant. Estimates will be interpreted in the context of effect sizes and CIs. Analyses will be performed using R version ≥4.3 (R Foundation for Statistical Computing, Vienna, Austria), SAS version 9.4 (SAS Institute, Cary, NC), or equivalent validated analytical platforms. No formal interim efficacy analyses are planned.

### Sample Size Estimation

The sample size was calculated on the basis of expected differences in arrhythmia‐free survival between the 2 treatment arms. Estimates will be interpreted in the context of CIs and effect size rather than significance alone. Prior data from a pilot study^55^ and published trials and analyses^5^, ^30^ were used to estimate procedural success rates, which were interpreted conservatively given the high‐risk nature of the study population. The sample size is based on a 1:1 randomized allocation to test the superiority of HA over CA. The power calculation assumed an anticipated incidence of sinus rhythm of 70% in the hybrid arm and 43% in the catheter arm. Using these numbers, with a standard α level of 0.05 and 80% power, 52 patients per arm (104 total) would be required to demonstrate the superiority of HA over CA. To account for potential dropout, loss to follow‐up, and estimation uncertainty, a 10% attrition rate and an additional 5% safety margin were applied. The final enrollment sample size was set at a minimum of 120 patients (60 per arm). The trial is powered for the primary efficacy end point; secondary and subgroup analyses are supportive and exploratory.

## Discussion

AF and HF with reduced LVEF represent 2 distinct but overlapping pathological processes, each presenting an escalating challenge to health care: AF is the most common sustained cardiac arrhythmia worldwide, affecting an estimated 53 million people in 2021, having risen in prevalence by 137% since 1990 and continuing to rise with the aging population.^56^ In parallel, HF is estimated to have affected 56 million people in 2019,^57^ with the prevalence doubling since 2010.^58^ AF confers significant morbidity and death, most notably by increasing the risk of stroke and HF,^59^ and HF represents an important public health concern due to high rates of death and rehospitalization. When both conditions coexist, their impact is magnified by the causal, synergistic, and reciprocal relationship between them. Prognostically, the development of either condition in the presence of the other is associated with poorer outcomes.^4^ AF occurs in 30% to 40% of people with acute and chronic HF and is diagnosed in more than half of patients with new‐onset HF.^60^ The onset of AF results in loss of coordinated atrial contraction, consequential irregular ventricular filling, and time‐related impairment of myocardial function. In conjunction with fast and erratic ventricular heart rates, the incidence of HF can increase 3‐fold.^61^ Similarly, the prevalence of HF increases with AF severity and may be found in 33% of paroxysmal patients and 56% of those with permanent AF.^62^ The impact of this convergence of pathology is striking. Patients with HF who develop AF exhibit a >2‐fold increased risk of death, and patients with AF who develop HF have a 29% increased risk of death.^63^

In settings where AF is the leading cause of LV dysfunction, durable restoration of sinus rhythm can significantly alter the clinical trajectory. Even in patients for whom the pathogenesis of HF is less well defined, the presence of AF is associated with a significantly worse prognosis.^64^ CASTLE‐AF demonstrated that CA reduces all‐cause death and HF hospitalizations while significantly improving LVEF.^3^ Similar findings are reported in a subanalysis of CABANA, which showed a 3.1% absolute risk reduction in all‐cause death^65^ and in a meta‐analysis of 2293 patients from 6 RCTs, which showed an absolute reduction of 5.19% in all‐cause death when compared with medical therapy.^66^ In such circumstances, AF ablation extends its role beyond symptom management to become a disease‐modifying strategy. However, restoring sinus rhythm remains a challenge in patients with persistent or long‐standing persistent AF: Conventional rhythm control with AADs or serial electrical cardioversions is limited by high relapse rates, side effects, and proarrhythmia, and outcomes from CA in persistent AF remain suboptimal. In clinical trials focusing on persistent AF (including those with HF), between 20% and 57% of patients require repeat ablation procedures within 1 to 2 years due to arrhythmia recurrence^25^ and meta‐analyses comprising >18 000 patients with persistent AF revealed that only 43% of patients remained in sinus rhythm at ≈2‐year follow‐up after a single procedure, improving to 69% with multiple procedures and AAD use.^5^

Emerging evidence suggests that a hybrid ablation approach results in superior maintenance of sinus rhythm compared with CA, an outcome particularly relevant for high‐risk cohorts such as those with HF. In the multicenter CONVERGE trial, convergent HA resulted in significantly higher freedom from recurrent atrial arrhythmia off AADs at 12 months (53.5% versus 32.0%; *P*=0.0013), increasing to 67.7% versus 50% on AADs.^30^ Similarly, in the single‐center HARTCAP‐AF (Hybrid Versus Catheter Ablation in Persistent Atrial Fibrillation) trial, freedom from recurrent arrhythmia at 12 months was higher after thoracoscopic HA off AADs (89% versus 41% following CA; *P*=0.002) and at 3 years (68.4% vs 22.7%; *P*=0.002).^25^, ^67^ Most recently, the international multicenter CEASE‐AF study confirmed the efficacy of a hybrid epicardial–endocardial approach, reporting higher arrhythmia‐free survival following HA at 12 months (71.6% versus 39.2% following CA) and at 24 months (66.3% versus 33.3% following CA; both *P*<0.001).^26^, ^27^ The mechanisms underlying the incremental benefit of epicardial ablation are not fully defined but likely reflect the limitations of endocardial‐only ablation in achieving durable and safe lesion formation,^6^, ^7^ as well as the inability to directly target epicardial substrate critical to the sustenance or initiation of AF in its more persistent or resistant forms through the increasingly recognized mechanism of endocardial–epicardial dissociation.^17^

Despite growing evidence supporting the superiority of HA over CA in persistent AF, evidence for its use in patients with reduced LVEF remains scarce. In the CONVERGE, HARTCAP‐AF, and CEASE‐AF trials, the mean LVEFs in the HA arm were 55.3±7.8%, 55.0±7.0%, and 58.3±9.0%, respectively. Among minimally invasive epicardial ablation strategies, the convergent technique may be most suitable for patients with HF, particularly when right ventricular function is also affected, as it does not require thoracoscopy and avoids the hemodynamic challenges and hypoxemia that may be associated with single lung ventilation.^68^, ^69^ However, only a single retrospective study reports the use of convergent HA in patients with reduced LVEF,^34^ with similar small retrospective‐only analyses following thoracoscopic HA reported.^70^ The paucity of evidence represents a significant research gap, given that patients with AF and reduced LVEF are most likely to benefit from durable maintenance of sinus rhythm and from the care provided by a multidisciplinary, collaborative AF management team, such as that offered with HA.^44^

HALT AF is designed to address these gaps and will provide level I evidence of the efficacy and safety of convergent HA in patients with reduced LV function. To our knowledge, our trial will be the first to directly compare outcomes in this cohort. By randomizing patients with persistent AF and impaired LVEF to strategically equivalent ablation strategies via HA or CA and by implementing rigorous, repeated rhythm surveillance with blinded adjudication and quantifying atrial and ventricular reverse remodeling alongside quality‐of‐life measures, HALT AF has been designed to offer mechanistic insights and help inform future practice guidelines and the selection of optimal rhythm‐control therapy for persistent AF, particularly in high‐risk populations. The trial was registered in June 2022 and randomized its first patient soon afterward. The trial duration is 12 months, with a planned 24‐month extension. The efficacy end point is freedom from atrial arrhythmia lasting >30 seconds, which remains the gold standard for AF ablation trials. Aligning with this outcome will enable comparability with historical and similar ablation trials. Acknowledging the long‐standing debate about its validity in the modern era, particularly in patients with impaired LVEF,^71^ important secondary end points will assess the impact of HA and CA on clinically relevant and dedicated HF outcomes, including key quality‐of‐life measures.

## Limitations

HALT AF is a pragmatic strategy trial evaluating outcomes following randomization to catheter or hybrid ablation, with efficacy analyses reflecting treatment allocation rather than procedural optimization, and follow‐up aligned with real‐world clinical pathways. The trial, therefore, assesses the consequences of selecting a hybrid versus catheter ablation strategy rather than attempting to harmonize inherently different procedures. Within this framework, the hybrid approach entails a greater up‐front procedural burden than single‐stage endocardial‐only CA and requires completion of both epicardial and endocardial components to achieve the intended electrophysiological end points (PVI and PWI). Differences in procedural burden, recovery, and cumulative exposure are intrinsic to the strategies being compared and are explicitly accounted for in the trial design and reporting framework. Interstage events are prospectively captured and incorporated into prespecified safety and secondary analyses. Participants who do not complete stage 2 remain included in the primary efficacy analysis according to prespecified end point definitions, with additional per‐protocol and sensitivity analyses assessing robustness.

Additional heterogeneity arises from the discretionary exclusion of LAA in the HA arm for thromboembolic risk management. LAA exclusion is neither protocol‐mandated nor randomized and is not intended as a rhythm‐control intervention. The effect of mechanical epicardial LAA exclusion on AF recurrence remains uncertain, and randomized studies evaluating comparable mechanical epicardial LAA exclusion strategies have not demonstrated consistent reductions in AF burden beyond conventional ablation.^72^ To ensure transparency, all LAA interventions will be prospectively recorded and reported. Rhythm outcomes will be presented descriptively by LAA‐exclusion status, with prespecified sensitivity analyses examining the consistency of strategy‐level findings; these analyses are exploratory. End point adjudication is blinded to procedural details, ensuring that LAA management does not influence event classification.

Similarly, endocardial ablation in either arm may be performed using RFA or PFA. HALT AF is designed to compare ablation strategies rather than ablation energy modalities and is not powered to compare endocardial ablation sources. To preserve internal validity, identical electrophysiological procedural end points (PVI and PWI) are mandated irrespective of energy source, ensuring that comparisons reflect ablation strategy (endocardial–epicardial versus endocardial) rather than technology. Thus far, randomized studies and meta‐analyses have not shown PFA to be superior to RFA in terms of freedom from arrhythmia.^47^, ^48^, ^49^ The inclusion of contemporary endocardial technologies enhances external validity and ensures that no participant is denied potentially beneficial or emerging therapies by virtue of trial enrollment. Acute procedural success will be reported to confirm end point achievement, and ablation energy use will be examined in prespecified exploratory analyses. Cryoablation was not included, given its limited role in contemporary PW ablation.

All procedures are performed at UK tertiary electrophysiology centers with expertise in advanced AF ablation. While this may limit generalizability to nonspecialist settings, it reflects current referral pathways and the infrastructure required to deliver HA safely. To permit a clear assessment of comparative effectiveness, patients with prior AF ablation were excluded; accordingly, the findings may not be directly generalizable to populations undergoing repeat ablation.

Finally, rhythm surveillance mirrors contemporary practice with intermittent monitoring. Continuous implantable monitoring was not mandated, as it would require an additional invasive procedure beyond routine care. In accordance with guideline recommendations for postablation monitoring in clinical trials, prolonged Holter monitoring was selected to enhance AF detection and quantification.^73^, ^74^ Implantable loop recorders have high rates of false‐positive alerts,^75^ with studies in patients following AF ablation reporting that intermittent prolonged Holter monitoring is comparable to continuous monitoring in detecting arrhythmia recurrence.^76^ While this may fail to capture brief or asymptomatic arrhythmia recurrences, persistent arrhythmia in patients with reduced LVEF is less likely to be asymptomatic, given that it may precipitate cardiovascular decompensation, and the clinical significance of asymptomatic atrial high‐rate episodes in this population remains uncertain.^1^, ^73^

## Conclusions

The coexistence of persistent AF and HF with reduced LVEF represents a formidable health care challenge, marked by cumulative increases in morbidity and death, diminished quality of life, and repeat interventions and hospitalizations. HALT AF will be the first randomized trial to directly compare convergent HA and CA in patients with persistent AF and reduced LVEF, targeting a clinically important, high‐risk, and understudied population with a substantial unmet need, in whom durable rhythm control may have meaningful clinical benefit. The findings will provide high‐level evidence to inform rhythm‐control strategies in persistent AF and HF and help define the role of convergent hybrid ablation in future guidelines and clinical practice.

## Sources of Funding

St George's Hospital, London, UK, is the sponsor of HALT AF. AtriCure provided limited support through a small educational grant. AtriCure has no involvement in the protocol or trial design, nor in the creation, planning, or conduct of the HALT AF study. AtriCure will have no access to any data generated and will not be involved in data analysis or the primary presentation of the study's outcomes.

## Disclosures

A.M. and R.A.K. report educational presentations for AtriCure. The remaining authors have no disclosures to report.

## Supporting information

CONSORT 2025 checklist
